# Ketogenic Diet as a Preventive and Supportive Care for COVID-19 Patients

**DOI:** 10.3390/nu13031004

**Published:** 2021-03-20

**Authors:** Elena Gangitano, Rossella Tozzi, Orietta Gandini, Mikiko Watanabe, Sabrina Basciani, Stefania Mariani, Andrea Lenzi, Lucio Gnessi, Carla Lubrano

**Affiliations:** 1 Department of Experimental Medicine, Sapienza University of Rome, 00161 Rome, Italy; mikiko.watanabe@uniroma1.it (M.W.); sabrinabasciani@yahoo.it (S.B.); s.mariani@uniroma1.it (S.M.); andrea.lenzi@uniroma1.it (A.L.); lucio.gnessi@uniroma1.it (L.G.); 2 Department of Molecular Medicine, Sapienza University of Rome, 00161 Rome, Italy; rossella.tozzi@uniroma1.it (R.T.); orietta.gandini@uniroma1.it (O.G.)

**Keywords:** SARS-CoV-2, COVID-19, obesity, ketogenic diet, VLCKD, inflammation, viral infections, respiratory failure

## Abstract

Severe obesity is associated with an increased risk of admission to intensive care units and need for invasive mechanical ventilation in patients with COVID-19. The association of obesity and COVID-19 prognosis may be related to many different factors, such as chronic systemic inflammation, the predisposition to severe respiratory conditions and viral infections. The ketogenic diet is an approach that can be extremely effective in reducing body weight and visceral fat in the short term, preserving the lean mass and reducing systemic inflammation. Therefore, it is a precious preventive measure for severely obese people and may be considered as an adjuvant therapy for patients with respiratory compromise.

## 1. Introduction

Coronavirus 2019 disease (COVID-19), caused by SARS-CoV-2 virus, has spread worldwide causing a pandemic since March 2020, now leading to new waves of infection. Overall fatality rate reached 2.3% [1] and, to date, 2,343,069 cases of COVID-19 and 80,253 (3.4%) deaths have been registered in Italy [2].

In most cases the clinical presentation is characterized by fever, dry cough, fatigue and mild pneumonia, although critical forms with desaturation and respiratory failure, septic shock, and/or multiple organ dysfunction can also occur; it has been estimated that moderate and severe forms can affect 14% and 5% of patients, respectively [1]. COVID-19 management consists of supportive therapy and preventing respiratory insufficiency through oxygen therapy or positive ventilation. The most widely adopted therapeutic protocol is based on the use of antibiotic prophylaxis, steroids and anticoagulant therapy, although there is no conclusive evidence supporting their role [3]. In order to limit the typical coagulative hyperactivation and the well-known condition of thrombosis susceptibility [4,5], heparin is now used in early stage COVID-19 patients; however, intensive care units are gradually filling up again, fearing the national health system collapse.

COVID-19 mortality is highly correlated to the severity of the inflammation-related cytokine storm and to the presence of multiple comorbidities (obesity, type 2 diabetes, hypertension, chronic obstructive pulmonary disease) increasing the risk of developing critical forms of infection [6]. In light of these considerations, it is therefore mandatory to pursue new strategies to reduce risk factors and to limit the development of the cytokine storm syndrome (CSS) in order to prevent patients’ worsening and access to emergency rooms.

The nutritional approach to COVID-19 patients is extremely important to ensure the correct amount of nutrients, necessary to face the infection and the body’s capacity to face and fight the virus. Current European Society for Clinical Nutrition and Metabolism (ESPEN) expert statements for COVID-19 patients recommend considering energy needs of 27–30 kcal per kg body weight and day, and 1–1.3 g per Kg of proteins, depending on disease status. Fat and carbohydrate ratio are currently suggested to be 30:70 for patients without respiratory deficiency and 50:50 for ventilated patients [7].

The ketogenic diet (KD), reducing carbohydrates oral intake, allows the hepatic production of ketone bodies and the onset of nutritional ketosis as a result of an increased utilization of fat as metabolic fuel when the availability of glucose is low. Ketone bodies are attracting more and more attention for their anti-inflammatory role and immune metabolism modulation [8]. Besides the well-known metabolic advantages (better hyperglycemia control, reduction of insulin resistance, improvement of hepatic steatosis), several “non-classical” beneficial effects have been attributed to KDs, including growth factors, leptin or IGF-1 modulation [9], together with the protection of renal, brain function and anti-viral effects [10].

KDs provide for a deprivation of carbohydrate content equal to 5–10% of total kcal daily intake, although the specific macronutrient composition may vary. As reported by Watanabe et al. [11], ketogenic diets differ mainly in calorie intake and protein content. High Fat Ketogenic Diets (HFKD) are characterized by a restriction of carbohydrates (CHO) < 50 g per day with unrestricted intake of fat, a relative increase of protein (0.8–1.2 g per day), and ad libitum caloric intake; very low-calorie ketogenic diets (VLCKD) are characterized by approximately the same amount of CHO and protein as in HFKDs, but significantly lower fat and therefore calorie intake, which goes as low as 600 kcal/daily. Very low-calorie diets (VLCD), providing a marked restriction of daily calorie intake, are characterized instead by a variable amount of carbohydrate intake which may or may not be able to induce ketosis [12] (Table 1).

While HFKDs are still used in refractory epilepsy in children, VLCKD are now recommended in severe or sarcopenic obesity, prior to bariatric surgery, to improve glycemic control, dyslipidemia and for a rapid reduction of cardiovascular risk factors in obese patients, not responsive to standard diets [12].

Current contraindications to the VLCKD include type 1 diabetes mellitus, kidney or liver failure, heart failure, cardiac arrhythmias, recent stroke, myocardial infarction, pregnancy and breastfeeding. Of note, active/severe infections and respiratory failure are currently among the conditions not recommended for implementing a VLCKD regimen for a hypothesized immunosuppression and acidosis risk, respectively [13]. Nevertheless, studies conducted in the past have reported good results, also highlighting some benefits derived from ketosis [13]. As per HFKDs, patients with CVD, heart, liver or kidney disease need close medical supervision in order to safely undergo such regimen, and those with severe dyslipidemia or a history of hypertriglyceridemia associated pancreatitis are recommended against undergoing this dietary regimen [14].

The aim of this work is to highlight the potential role of KDs in the management and prevention of COVID-19, focusing on the beneficial effects that may exert on inflammation, immune system and respiratory function.

## 2. Low Chronic Inflammation, COVID-19 and Ketogenic Diet

As described above, severe forms of COVID-19 are characterized by an ineffective adaptive immune response that leads to a persistence in C-reactive protein (CRP) and interleukin (IL) -6 elevation [15]. This pattern falls within the so-called chronic low-grade inflammatory phenotype (CLIP), a phenomenon that underlies many of the diseases associated with more critical forms of COVID-19, such as diabetes, obesity, insulin-resistance, hypertension and atherosclerosis [16]. All these metabolic derangements are closely related to inflammation triggered by the abnormal expansion of visceral adipose tissue, which has been shown to predict poor COVID-19 prognosis as well as respiratory indicators [17]. Specifically, the white adipose tissue M1 macrophages secretion of pro-inflammatory cytokines including tumor necrosis factor (TNF) alpha, IL-6, CRP, IL-1, is increased, whereas a steep decline occurs in the production of anti-inflammatory cytokines like IL-10, the interleukin-1 receptor antagonist (IL-1RA), and adiponectin. Not only the adipose tissue, but also the immune cells, liver, brain, muscles and pancreas suffer from the inflammatory insult in subjects with obesity. Macrophage-like Kupffer cells initiate the inflammatory process in the liver preceding the inflammatory signals produced by the white adipose tissue, which may further lead to hepatic-necro-inflammation [18]. Moreover, role of P-loop domain belonging to the STAND class of NTPases with homology to the oligomerization module found in AAA+ ATPases (NACHT), Leucine-rich repeat (LRR), and NOD-like receptors (NLRs) Pyrin Domain-Containing 3 Protein (NLRP3) for maintenance of chronic inflammation is crucial. In fact, in response to activation of innate immune receptors by stimuli such as microbial ligands, transcription of pro-inflammatory genes, including those encoding NLRP3 and pro-IL1β, is induced [19].

KDs inhibit aerobic glycolysis, which has been proven to occur following inflammatory activation of cells from both myeloid and lymphoid lineage; in particular, KDs prevent the differentiation and effector functions of inflammatory cells, while promoting the differentiation of regulatory subsets. Moreover, the ketone body β- hydroxybutyrate blocks NLRP3 inflammasome activation [20].

## 3. Immune System, COVID-19 and Ketogenic Diet

SARS-CoV-2 infects lung cells and enters host epithelial cells through Transmembrane Serine Protease 2 (TMPRSS2) action and spike protein binding Angiotensin Converting Enzyme 2 (ACE-2) receptor. After alveolar epithelial cells pyroptosis-induced death and damage-associated molecular patterns (DAMPs) release, macrophages and monocytes are recruited and cytokines secreted. More specifically, in case of a dysfunctional immune response, we observe an abnormal monocytes, macrophages and T-cells infiltration favored by vascular permeability, a systemic cytokine storm (IL-6, IFN gamma, IL-2, IL-10, Granulocyte colony-stimulating factor G-CSF, TNF), clinical worsening (pulmonary oedema and pneumonia) and widespread inflammation and/or multiorgan damage due to excessive TNF and reactive oxygen species (ROS) production. On the contrary, in a healthy immune system, initial inflammation attracts virus specific T-cells to the site of infection, where they can eliminate the infected cells before the virus spreads. Neutralizing antibodies in these individuals can block viral infection resulting in early recovery [15]. Noteworthy, viral infection can also result in an aberrant cytokine production by the immune cells such as monocytes and macrophages. Elderly people seem to be more susceptible to critical forms of COVID-19 due to an ageing lung microenvironment causing altered dendritic cell maturation and migration to the lymphoid organs and to an inefficient IFN response [21].

Karagiannis et al. [22] demonstrated that restricting dietary glucose by feeding mice a HFKD (72% fat, 2.4% sugar) largely ablates lung-resident type 2 Innate Lymphoid Cells (ILC-2) and reduces airway inflammation by impairing fatty acid metabolism and the formation of lipid droplets. Chronic activation of ILCs, typical of allergenic airway inflammation, needs exogenous fatty acids which are transiently stored in lipid droplets and therefore converted into phospholipids to promote ILCs proliferation. This metabolic program, imprinted by IL-33 and regulated by the genes Peroxisome proliferator-activated receptor gamma (PPAR-γ) and Diacylglycerol O-Acyltransferase 1 (Dgat1), is controlled by glucose availability as well as mammalian target of rapamycin (mTOR) signaling. Moreover, Goldberg et al. reported that a HFKD allows for better survival and increased protective IL-17-secreting γδ T cells in the lungs of mice with influenza virus [10], while Ryu et al. have recently provided preclinical evidence that a HFKD is capable of providing a protective effect against the animal equivalent of COVID-19 in aged mice, with the maintenance of a better oxygen saturation and an increase in γδ T cells [23].

## 4. Obesity, Viral Infections and Respiratory Function

Weight excess is associated with a higher susceptibility to viral infections [3], as seasonal and H1N1 influenza [24,25], and a higher risk of hospitalization for these conditions [26,27,28,29]. In recent years, during the H1N1 influenza pandemic, obesity has been shown to be associated with hospitalization and death [29] and critically ill patients were frequently morbidly obese [25]. Similarly to other viral infections, severe obesity is associated with a high risk of COVID-19 complications [30]. Among obesity comorbidities, hypertension, dyslipidemia, prediabetes and insulin resistance might predispose individuals to cardiovascular events and increased susceptibility to infection via atherosclerosis. Resulting cardiac dysfunction and kidney failure can more easily lead to pneumonia-associated organ failures [31]. Moreover, visceral adipose tissue—a reliable and specific marker of insulin resistance—has been independently associated with the need of intensive care unit (ICU) resulting as the strongest predictor of worse prognosis in patients with COVID-19 [17]. Considered this, a nutritional approach that can break down insulin resistance such a HFKD, might have beneficial implications in COVID-19 prognosis likely without any detrimental effects.

Obese patients are predisposed to the development of chronic and acute respiratory illnesses [32,33], including respiratory tract infections [34]. The reasons for this susceptibility to respiratory disease are many and not completely elucidated yet [35,36]. Obese people have alterations in respiratory physiology [37] and immune response [24,33] and, consequently, develop a lower response to antiviral therapies and vaccinations [24]. The alterations in respiratory physiology consist in a decreased functional residual capacity and reduced expiratory reserve volume, hypoxemia and ventilation perfusion abnormalities [28,37]. The presence of Obstructive Sleep Apnea Syndrome (OSAS), which is common in obese people, may predispose the patients to COVID-19 complications [38].

Obesity is characterized by low-grade systemic inflammation, that may be related to the pathogenesis of respiratory conditions [33]. Fat tissue may accumulate within the lungs, as observed in the airways of obese humans [39] and in the alveolar interstitium of obese diabetic rats [40]. Adipose tissue accumulation in the outer wall of large airways positively correlated with inflammatory infiltrate of eosinophils and neutrophils in patients with fatal asthma [39].

Animal models of obesity showed that during influenza infection there is increased lung permeability, leading to protein leakage into the bronchoalveolar lavage fluid. For the resolution of the infection, the repair of the damaged epithelial surface is required, but wound repair is impaired. Increased lung oedema and oxidative stress have been observed as well [24].

There is evidence that immune system functioning is altered in obesity. T-cells diversity is reduced and this may be related to the T-cells poor response to influenza virus [24]. CD8+ T memory cells has been shown to be impaired, with consequent exacerbates lung complications and mortality [33]. These cells are responsible for an efficient immune response to vaccination [33], with consequent reduced response to vaccination in obese people [24]. Moreover, obesity may be a factor that exacerbates the aging of the immune system [24].

In addition, the high ACE-2 expression in adipose tissue may play a role in obese patients’ susceptibility to COVID-19 infection, since SARS-CoV-2 shows high affinity for this enzyme [41].

Therefore, interventions aimed to weight loss in obese patients are warranted to prevent viral infection susceptibility and their complications and theoretically may ameliorate respiratory function.

## 5. Low-Carbohydrate Ketogenic Diets and Respiratory Function

VLCKDs are, to date, contraindicated for obese patients with respiratory failure [12]. However, some studies reported some beneficial effects from high-fat low-carbohydrate diets and detrimental effects of carbohydrate loads on respiratory parameters. These studies, anyway, often did not specify if patients were in ketosis, but used low amount of CHO, possibly leading to ketosis.

Two studies on a total of 40 healthy patients [42,43] reported that a VLCKD (848 kcal/day; protein: carbohydrate: fat = 43:14:43%) and a HFKD (10% calories from carbohydrate) diet reduced CO_2_ output without modifying oxygen uptake. Moreover, Rubini et al. compared a VLCKD regimen to a hypocaloric Mediterranean diet showing that only the VLCKD significantly decreased respiratory exchange ratio (*p* < 0.05) in addition to higher fat mass loss in healthy patients. Therefore, these diets may be helpful in respiratory patients for reducing CO_2_ body stores levels and dyspnea at rest. On the other hand, a study on 17 healthy women who were administered a HFKD (2400 kcal/day), reported earlier muscle fatigue during daily life activities [44].

Chronic Obstructive Pulmonary Disease (COPD) is often accompanied with hypercapnia and hypoxemia. A reduction in carbon dioxide production would reduce the workload of respiratory muscles and therefore be beneficial for these patients. Some studies focused on the administration of HFKD in COPD patients, and beneficial or, at least, neutral results were observed.

In twelve clinically stable COPD patients, the administration of a high-fat meal had a small effect on gas exchange parameters compared to 12 healthy controls, whereas a high-carbohydrate diet was detrimental on gas exchange parameters, especially in COPD patients [45]. No differences in pulmonary function were detected in 36 COPD patients comparing the administration of a moderate-fat meal with a high-fat meal [46]. On the other hand, the administration of a HFKD in COPD patients with hypercapnia led to an amelioration of respiratory parameters in an overall sample of 74 underweight patients [47,48].

In patients with respiratory failure, providing an adequate protein intake is extremely important to preserve skeletal muscle mass and function [7]. A high-fat low-carbohydrate diet has been reported as a potential useful tool to ameliorate respiratory failure [49,50,51].

In the literature, there are some evidences of a beneficial effect of a high-fat low-carbohydrate diet in mechanically ventilated patients [52,53,54], since it was able to reduce PaCO_2_ levels [52,53,55] and the time of mechanical ventilation [52,53].

## 6. COVID-19, Lockdown and KDs

Both HFKD and VLCKD represent valuable treatments despite being characterized by the presence of contraindications and capable of causing side effects. Therefore, they should be followed under strict medical supervision and be considered similar to pharmacologic treatment. A concern may be that during the isolation imposed during the pandemic, it is difficult to monitor a patient on the ketogenic diet undergoing rehabilitation. Just a few studies reporting the administration of a ketogenic diet during this pandemic have been published, and to the best of our knowledge none published results on its use in COVID-19 infected and/or respiratory patients yet.

Kossof et al. [56] administered a HFKD to patients with uncontrolled seizures, mainly children, during the pandemic, using a combined approach with in person meetings and telemedicine. The authors and the other members of the International Ketogenic Diet Study Group, pediatric consensus group, reported no issues regarding the maintenance of ketosis and seizure control in their group, and raised no questions about the safety of the ketogenic diet in case of respiratory infection. A similar approach in similar setting was used by Ferraris et al. [57] and no major issues were reported, but they did not specify if any of their patients was infected by COVID-19.

Soliman et al. [58] proposed the use of a ketogenic diet and intermittent fasting, with administration of medium-chain triglycerides, as a prophylactic measure and an adjuvant therapy for COVID-19. In fact many viruses, as the varicella-zoster [59], the cytomegalovirus [60] and the hepatitis C [61], need the fatty acid metabolism pathway for their replication, therefore the diet-induced metabolic switch leading to a reduction in the fatty acid synthesis pathways may help in reducing viral replication [58].

## 7. Conclusion and Future Perspective

### 7.1. KDs in COVID-19 Prevention

Obesity, and in particular visceral abdominal fat, has been indicated as an independent risk factor for worse prognosis in COVID-19, often associated with the need for intensive care [17,30,41,62]. These may be due to the impaired respiratory mechanics, increased airway resistance and impaired gas exchange [25,28,54], as well as obesity-related comorbidities [63], which appear to be directly related to the onset of complications and severe course of COVID-19. In particular, OSAS [38], metabolic syndrome, hypertension, Non-Alcoholic Fatty Liver Disease (NAFLD) and diabetes or insulin resistance have all shown to affect COVID-19 outcome negatively [55,56,57,58]. Finally, it should not be overlooked that obesity is associated with low chronic inflammation within a state of immunological dysfunction that can lead to increased risk of allergies [64] or ineffective response against infections [35] and vaccines [65].

KDs, and specifically VLCKDs, demonstrated to induce weight loss and diabetes remission. VLCKDs are currently used in bariatric surgery preparation [12] thanks to the ability in reducing hepatic volume [11] with a subsequent improvement in intra and post-operative care. Recent findings underlined immune advantages derived from ketone bodies, such as blockage NLRP3 inflammasome [20], reduction in chronic activation of ILCs and induction of protective γδ T-cells against infections [10]. Taken together, in addition to the benefit of airway inflammation prevention by impairing the formation of lipid droplets [22], KDs could be an excellent tool to prevent the infection and stem the damage induced by COVID-19 in the fragile population affected from obesity.

### 7.2. KDs in Supportive Care of COVID-19

Studies conducted in mice highlighted the beneficial effect of HFD- induced ketone bodies in COVID-19 models [10,23]. In humans, HFKDs has been experimented in Intensive Care Units (ICU) and good results have been reported in mechanically ventilated patients [52,55]. Moreover, telemedicine achieved good results in pediatric epileptic patients under HFKDs, either for safety and compliance, proving that it can be a valid tool to be adopted even in the event of quarantine and fiduciary isolation. On the basis of these considerations, several authors proposed KDs in COVID-19 management and some clinical trials are ongoing [66,67].

### 7.3. KDs during Rehabilitation Post SARS-CoV-2 Infection

Patients affected from COVID-19, especially elderly ones, often require ICU for a longer period (up to 20 days) than other more typical uses of ICU. Among Post Intensive Care Syndrome (PICS), impaired exercise tolerance, neuropathies, muscle weakness/paresis, severe fatigue are responsible for decreased exercise capacity, disability and compromised quality of life for months, even years after intensive care [68]. Muscle atrophy, as well as obesity and immune dysregulation, is associated with Growth Hormone/Insulin-like Growth Factor 1 (GH/IGF-1) impaired axis and might be a link between IGF-1 downregulation and COVID-19 severity [69]. Preserving muscle mass is essential in order to improve rehabilitation and to reduce costs for recovering people.

VLCKDs preserved muscle mass in obese patients [70,71] when a protein intake of at least 1.2 gr of protein/Kg was ensured; the same results have been confirmed when isocaloric KDs have been used in patients affected from multiple sclerosis, reporting a superiority compared to Mediterranean diet [72]. Furthermore, HFKD (75–80% calories from fat, carbohydrates <50 g per day and <10 g per meal) improves quality of life, lean mass and metabolic parameters (included IGF-1) in oncologic patients, compared to standard diet [73].

**Figure 1:**
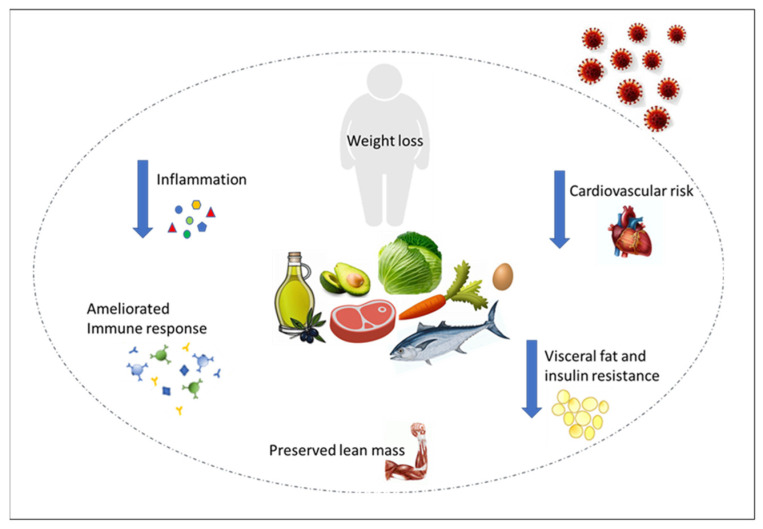
Mechanisms through which VLCKD with its consequent weight loss may reduce the susceptibility to severe SARS-CoV-2 infection and stem the damage induced by the virus.

In conclusion, VLCKDs administration might be considered in severely obese patients as an effective adjuvant therapy for COVID-19, first of all as a preventive measure, to achieve a fast weight loss [67], and secondly as an adjuvant therapy during rehabilitation (see Figure 1). More challenging is the hypothesis of administering HFKD during hospitalization or even more in delicate settings such as an intensive care unit or during positive ventilation; although several data support the evidence that limiting carbohydrate intake and promoting ketone formation may be helpful in ameliorating respiratory parameters. Furthermore, as extensively discussed, HFKDs show a strong anti-inflammatory effect and some data suggest that they may be useful for reducing viral replication. However, many studies are old, the samples small, and the ketosis not specifically addressed, therefore new clinical trials are needed. Hoping that the promising results observed in animal studies can be passed on to humans, we herein suggest considering KDs as an option to be considered for COVID-19 management within the current indications.

## Figures and Tables

**Table 1:** Main differences between ketogenic and low-carbohydrate diets (with the kind permission of Watanabe et al. [11]).

	Kcal/Day	CHO/Day	Fat/Day	Ketosis
High fat ketogenic diet (HFKD)	Usually unrestricted	<20–50 g	Unrestricted	Yes
Very low-calorie ketogenic diet (VLCKD)	<800 kcal	<20–50 g	Low	Yes
Very low-calorie diet (VLCD)	<800 kcal	<20–50 g	Low	Usually not
Low carbohydrate diet (LCD)	Variable	<130 g	Low	No

## Data Availability

Data sharing not applicable
